# Influence of Approach Velocity and Mesh Size on the Entrainment and Contact of a Lowland River Fish Assemblage at a Screened Irrigation Pump

**DOI:** 10.1371/journal.pone.0067026

**Published:** 2013-06-20

**Authors:** Craig A. Boys, Wayne Robinson, Lee J. Baumgartner, Ben Rampano, Michael Lowry

**Affiliations:** 1 Fisheries NSW, Port Stephens Fisheries Institute, Nelson Bay, New South Wales, Australia; 2 Fisheries NSW, Narrandera Fisheries Centre, Narrandera, New South Wales, Australia; 3 Charles Sturt University, Thurgoona, New South Wales, Australia; Aristotle University of Thessaloniki, Greece

## Abstract

Fish screens can help prevent the entrainment or injury of fish at irrigation diversions, but only when designed appropriately. Design criteria cannot simply be transferred between sites or pump systems and need to be developed using an evidence-based approach with the needs of local species in mind. Laboratory testing is typically used to quantify fish responses at intake screens, but often limits the number of species that can studied and creates artificial conditions not directly applicable to screens in the wild. In this study a field-based approach was used to assess the appropriateness of different screen design attributes for the protection of a lowland river fish assemblage at an experimental irrigation pump. Direct netting of entrained fish was used along with sonar technology to quantify the probability of screen contact for a Murray-Darling Basin (Australia) fish species. Two approach velocities (0.1 and 0.5 m.sec^−1^) and different sizes of woven mesh (5, 10 and 20 mm) were evaluated. Smaller fish (<150 mm) in the assemblage were significantly more susceptible to entrainment and screen contact, especially at higher approach velocities. Mesh size appeared to have little impact on screen contact and entrainment, suggesting that approach velocity rather than mesh size is likely to be the primary consideration when developing screens. Until the effects of screen contacts on injury and survival of these species are better understood, it is recommended that approach velocities not exceed 0.1 m.sec^−1^ when the desire is to protect the largest range of species and size classes for lowland river fish assemblages in the Murray-Darling Basin. The field method tested proved to be a useful approach that could compliment laboratory studies to refine fish screen design and facilitate field validation.

## Introduction

Irrigated agriculture accounts for 70% of the water withdrawn from freshwater systems by humans throughout the world and significant increases in the amount of water diverted from rivers occurred during the second half of last century [1]. Much of this growth has been in developing countries that have relatively large agricultural demands and a reliance on intensive dryland cropping [2]. Whilst water diversion has led to increased food security and economic gains, there has been an environmental cost [1]. The regulation and diversion of river flows required to facilitate dryland cropping can impact on fish by altering habitats and disrupting flow-dependent life history traits such as spawning and recruitment [3]–[5].

Physical removal of fish from rivers through entrainment at irrigation diversions has also been implicated in worldwide species declines [6], [7]. Mechanical injury and death can occur to fish that pass through diversion structures such as pumps and regulators [8], although many individuals do manage to survive diversion and form viable populations in off-river canals and impoundments [8], [9]. However, once diverted from the river there is seldom return passage and fish are lost from natural river populations [8], [10].

The loss of fish at irrigation diversions is an environmental problem with a solution. In many countries, irrigators and environmental agencies utilise fish screens on diversion points to decrease the numbers of fish being entrained without compromising the delivery of water to where it is needed [11]. The success of screening programs has been based upon having well-developed guidelines on screen design, that provide guidance on maximum velocities in front of the screen and the types of material that screens are made from [12]. Irrigation screening legislation exists in North America [13], [14], New Zealand [15], United Kingdom, Ireland, Switzerland, Netherlands, Denmark [16] and France [17]. Most fish screen guidelines in these countries (except New Zealand) were developed to protect migratory salmonids during seaward migration phases [11], [18]. It is increasingly accepted that other species impacted by diversions have been poorly considered in screen guidelines [19] and it is important to consider larger components of the migratory fish assemblage when developing guidelines [12].

In large floodplain rivers throughout South-East Asia, South America and Australia, a large proportion of the fish community (including large and small-bodied fish and their life history intervals) is migratory and requires protection at diversions [3], [20]–[24]. This poses a unique challenge for developing a screening program in these regions, as design criteria need to be developed for multiple species, size classes and development intervals. Much of the laboratory work undertaken to date to develop fish screening guidelines has focused on one or two species and few age classes (e.g. [25]–[27]), limiting the development of design criteria for a diverse assemblage of species. Furthermore, although laboratory studies can improve the understanding of species-specific swimming performance when exposed to different velocities [25], [26], or to quantify behaviour, injury and mortality of fish exposed to different screen conditions [25], [27]–[29], it remains difficult to replicate natural conditions or provide an accurate representation of fish encountering screens in the wild.

Field-based investigations of fish encountering screens are rare (but see Rose *et al.* [30]) possibly because high turbidity in many lowland river systems limits opportunities for direct observations [31]. The recent application of dual-frequency identification sonar (DIDSON) to fisheries research has proven to be an effective tool for quantifying fish abundance, size, behaviour and habitat use in dark or turbid waters, where traditional video capture techniques are ineffective [32]–[34]. Such technology may provide a powerful tool in studying fish behaviour around diversion screens without the need for direct interaction with fish and it could allow screen impingement rates to be quantified in a more natural environment.

In this study a field experiment at a simulated intake screen was used to test a variety of approach velocities (velocities 8 cm in front of and perpendicular to the screen face) and screening materials on the entrainment of a lowland river fish assemblage at water diversion. Because a fish screen may prevent entrainment, but injure or kill fish when they contact the screen face [35], we also directly observed fish behaviour in close proximity to the screen using DIDSON to quantify contact rates. This study provides the first data for the development of fish screen design criteria for Australian rivers and, to our knowledge, is the first study to take an assemblage-based approach to investigating the interaction between fish and an irrigation diversion screen in a riverine setting.

## Results

### Fish Entrainment

**Figure 1:**
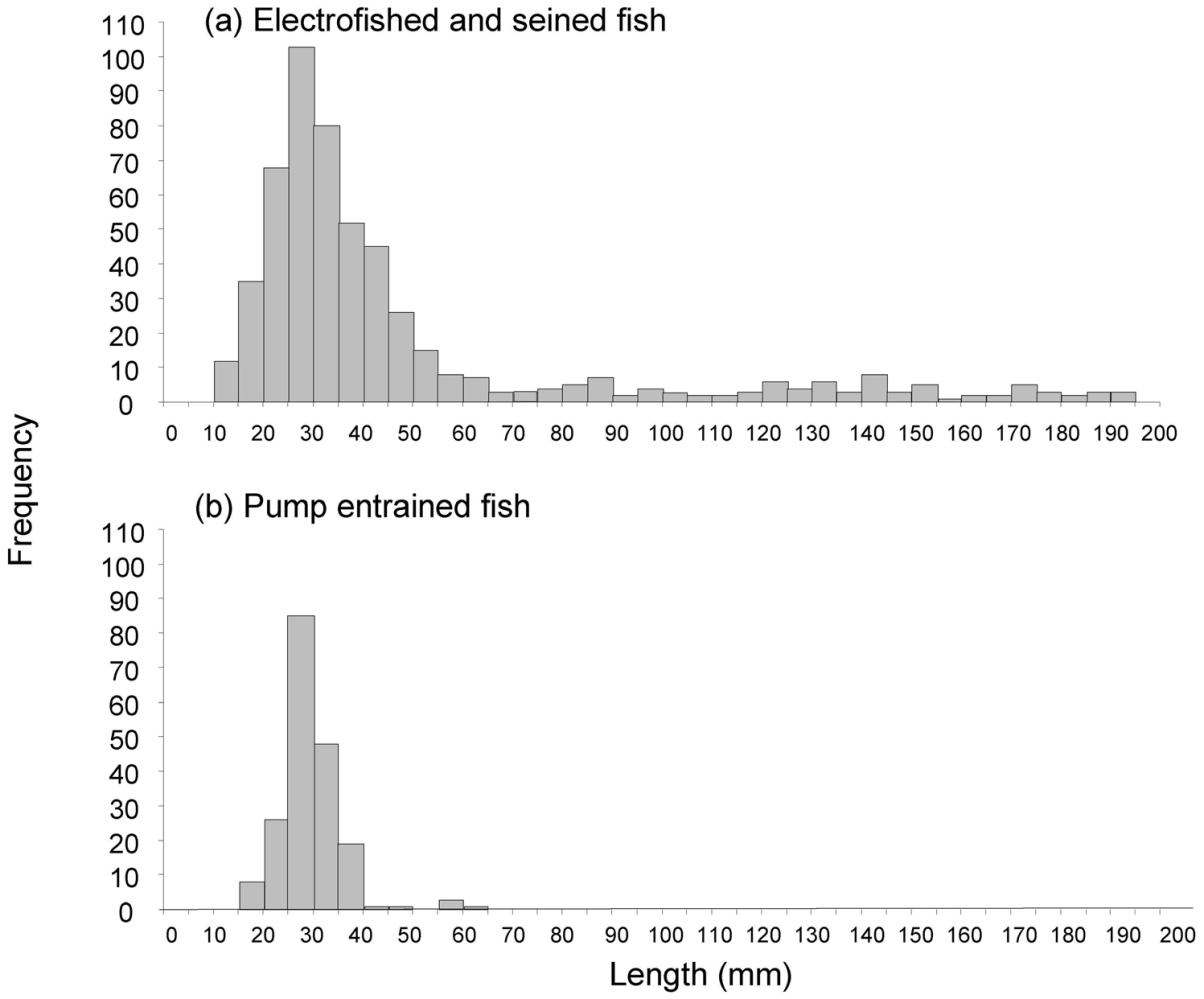
Length frequency plot for fish sampled a) by electrofishing and seine netting at pumping sites, and b) those collected after being entrained by the pump (all treatments pooled).

The fish assemblage within the vicinity of the experimental pumping station (obtained by electrofishing) comprised twelve species and encompassed all species still known to occupy lowland reaches of the Namoi River [36]. Only five of these species were entrained into the experimental pump system (Table 1). The assemblage Carp gudgeon (*Hypseleotris* spp.) were the most abundantly entrained (n = 138), followed by Australian smelt (*Retropinna semoni*) (n = 29). Spangled perch (*Leiopotherapon unicolour*) (n = 8), bony herring (*Nematalosa erebi*) (n = 2) and eastern gambusia (*Gambusia holbrooki*) (n = 1) were occasionally entrained in low abundances. Although a large size range of fish was sampled at the pumping sites, the catches were dominated by smaller fish (<60 mm length) and it was this size class that was most susceptible to entrainment (Figure 1).

**Table 1:** Number of fish entrained within the experimental pump system.

Common name	Scientific name	Electrofishing/Seine	Entrained by pump										
			0.1 m/s					0.5 m/s					Grand total
			no mesh	5 mm	10 mm	20 mm	total	no mesh	5 mm	10 mm	20 mm	total	pump
Goldfish	*Carassius auratus*	5	0	0	0	0	0	0	0	0	0	0	**0**
Flyspecked hardyhead	*Craterocephalus stercusmuscarum fulvus*	8	0	0	0	0	0	0	0	0	0	0	**0**
Common carp	*Cyprinus carpio*	102	0	0	0	0	0	0	0	0	0	0	**0**
Eastern gambusia	*Gambusia holbrooki*	28	0	0	0	0	0	0	1	0	0	1	**1**
Carp gudgeon	*Hypseleotris spp.*	200	7	0	7	1	15	38	38	11	36	123	**138**
Spangled perch	*Leiopotherapon unicolor*	24	0	0	0	0	0	5	0	2	1	8	**8**
Golden perch	*Macquaria ambigua*	26	0	0	0	0	0	0	0	0	0	0	**0**
Murray cod	*Maccullochella peelii peelii*	45	0	0	0	0	0	0	0	0	0	0	**0**
Murray-Darling rainbowfish	*Melanotaenia fluviatilis*	92	0	0	0	0	0	0	0	0	0	0	**0**
Bony herring	*Nematalosa erebi*	268	2	0	0	0	2	0	0	0	0	0	**2**
Australian smelt	*Retropinna semoni*	37	1	0	0	0	1	2	2	14	10	28	**29**
Freshwater catfish	*Tandanus tandanus*	2	0	0	0	0	0	0	0	0	0	0	**0**
	**Total**	**837**	**10**	**0**	**7**	**1**	**18**	**45**	**41**	**27**	**47**	**160**	**178**

Catches are pooled within each velocity and mesh combination. The electrofishing/seine column demonstrates the composition and relative abundance of fish captured at all the experimental site using electrofishing and seine netting.

**Figure 2:**
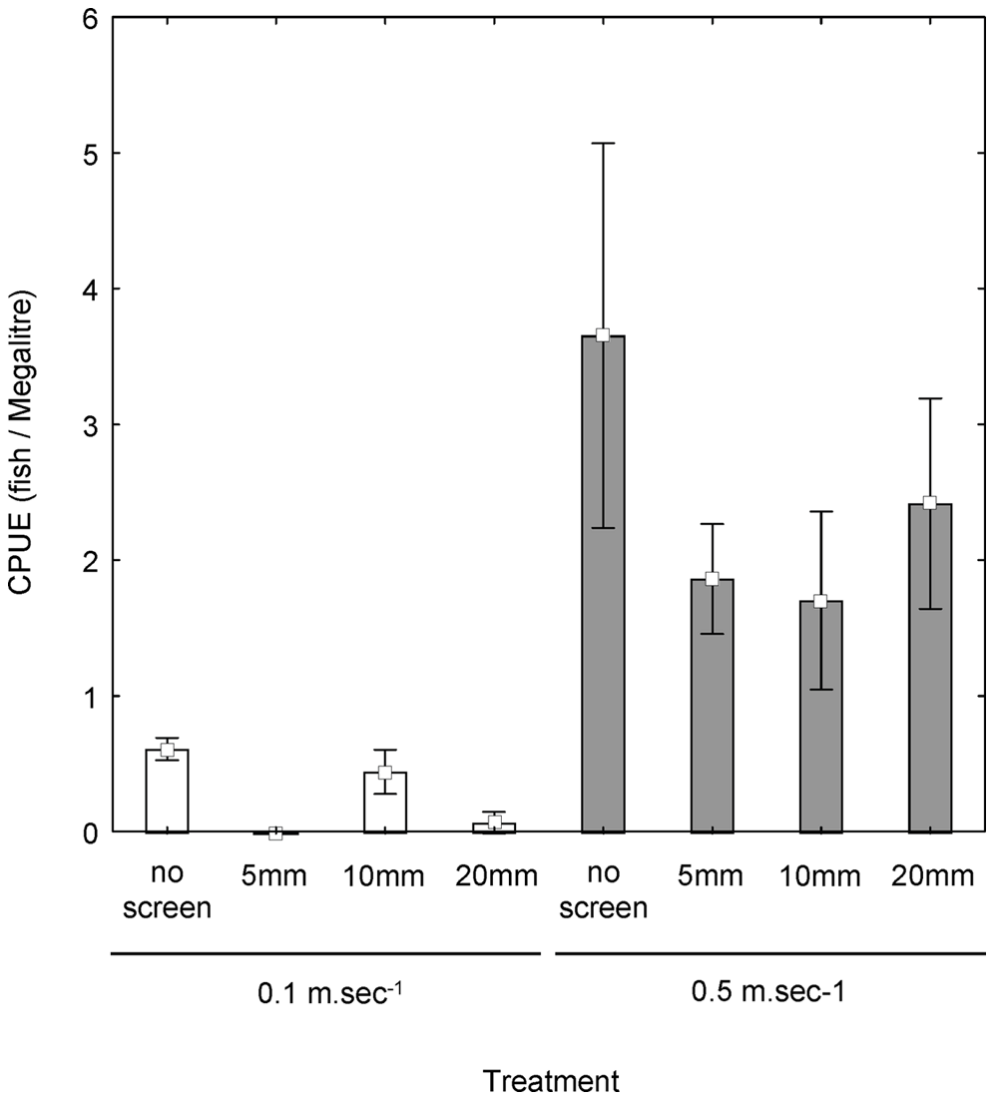
Mean (± S.E.) Cath per unit effort (number of fish/Megalitre) across different mesh and velocity treatments.

There was no effect of mesh size on the number of fish.ML^−1^ entrained at 0.1 m.sec^−1^ (F = 2.00, df = 3,6 p = 0.21) or 0.5 m.sec^−1^ (F = 1.29, df = 3, 6, p = 0.36) (Figure 2). Significantly more fish were entrained at 0.5 m.sec^−1^ than at 0.1 m.sec^−1^ (Figure 2) (F = 12.90, df = 1, 16, p<0.01). There were no significant interactive effects between velocity and mesh size on fish entrained (F = 0.60, df = 3,16, p = 0.630). Higher numbers of carp gudgeon, Australian smelt and spangled perch were entrained at increased approach velocities (Table 1).

### Screen Contact

Sonar revealed that fish were significantly more likely to contact the experimental screen at 0.5 m.sec^−1^ than at 0.1 m.sec^−1^ velocity (Likelihood ratio χ = 80.49, df = 1, p<0.0001). At 0.5 m.sec^−1^ 143 of the 436 (33%) fish observed contacted the screen compared to 259 of the 1902 (14%) fish observed at 0.1 m.sec^−1^. There was no difference in screen contact probability among the different mesh treatments at 0.5 m.sec^−1^ (χ^2^ = 4.7, df = 3, p = 0.198), but there was at 0.1 m.sec^−1^ (χ^2^ = 15.8, df = 3, p<0.01). At 0.1 m.sec^−1^ fish approaching the 5 mm mesh were 71% more likely to make contact than in the ‘no mesh’ control (Table 2). No significant difference was detected between the ‘no mesh’ control and 10 mm and 20 mm meshes (Table 2).

**Table 2:** Odds ratios for probability of screen contact for different mesh sizes when compared to the no mesh treatment at 0.1 m.sec^−1.^

Mesh size comparison withthe no screen treatment	Odds Ratio*	Confidence interval	Significance
5 mm	1.71	1.1 - 2.8	<0.05
10 mm	0.62	0.4 - 1.1	ns
20 mm	1.36	0.8 - 2.2	ns

* The odds ratio is the increase or decrease in the probability of contact when compared to the ‘no mesh’ treatment. For example, at 10 mm mesh size the probability of screen contact is 1÷0.62 = 1.61. Therefore contact is 61% less likely using the 10 mm mesh than the ‘no mesh’ treatment, however this was non-significantly (ns) different than a 1∶1 ratio at the p = 0.05 level.

**Figure 3:**
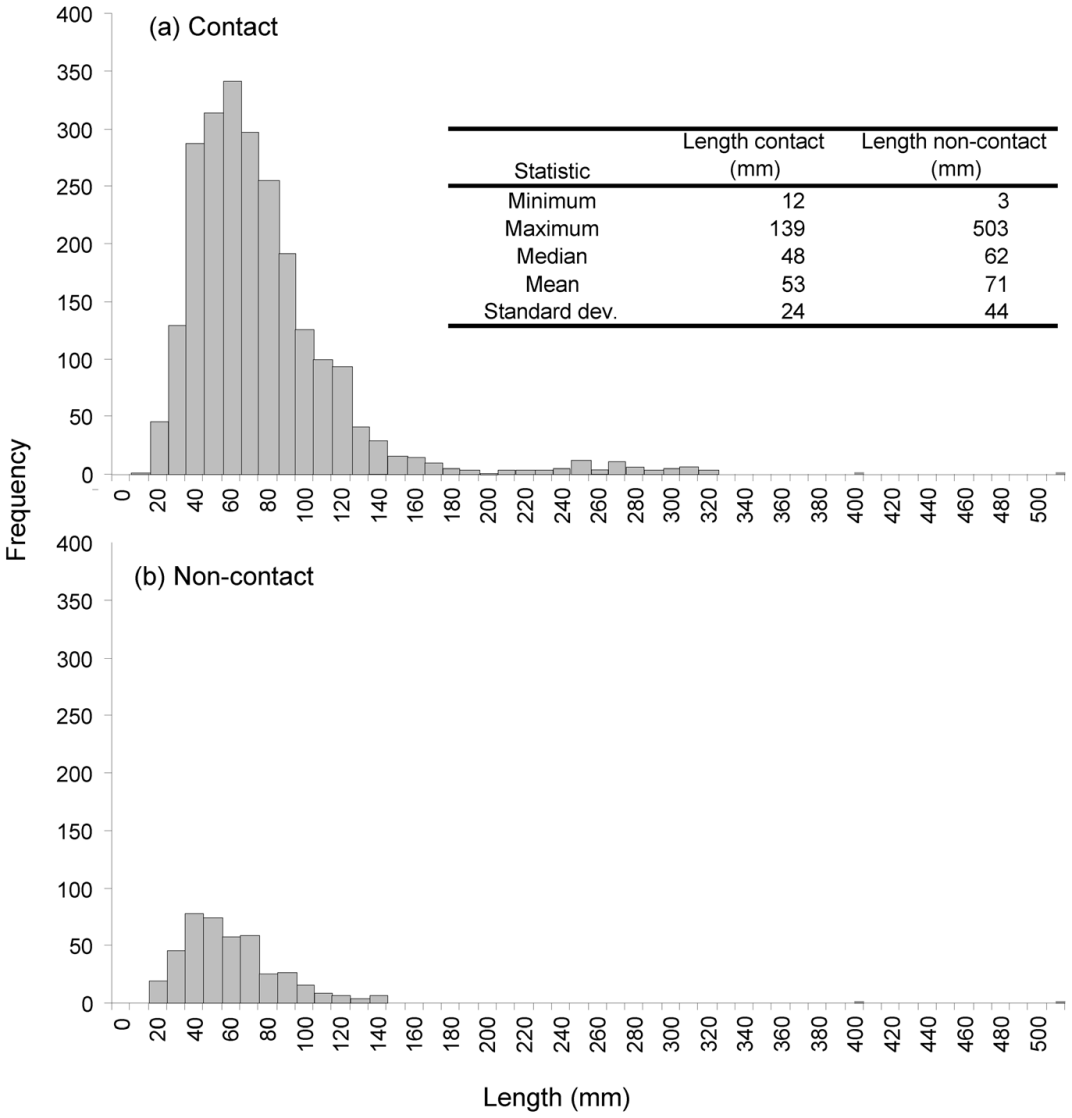
Length frequency plot showing the size range of fish observed by sonar to make a) contact or b) avoid contact with the experimental screen.

**Figure 4:**
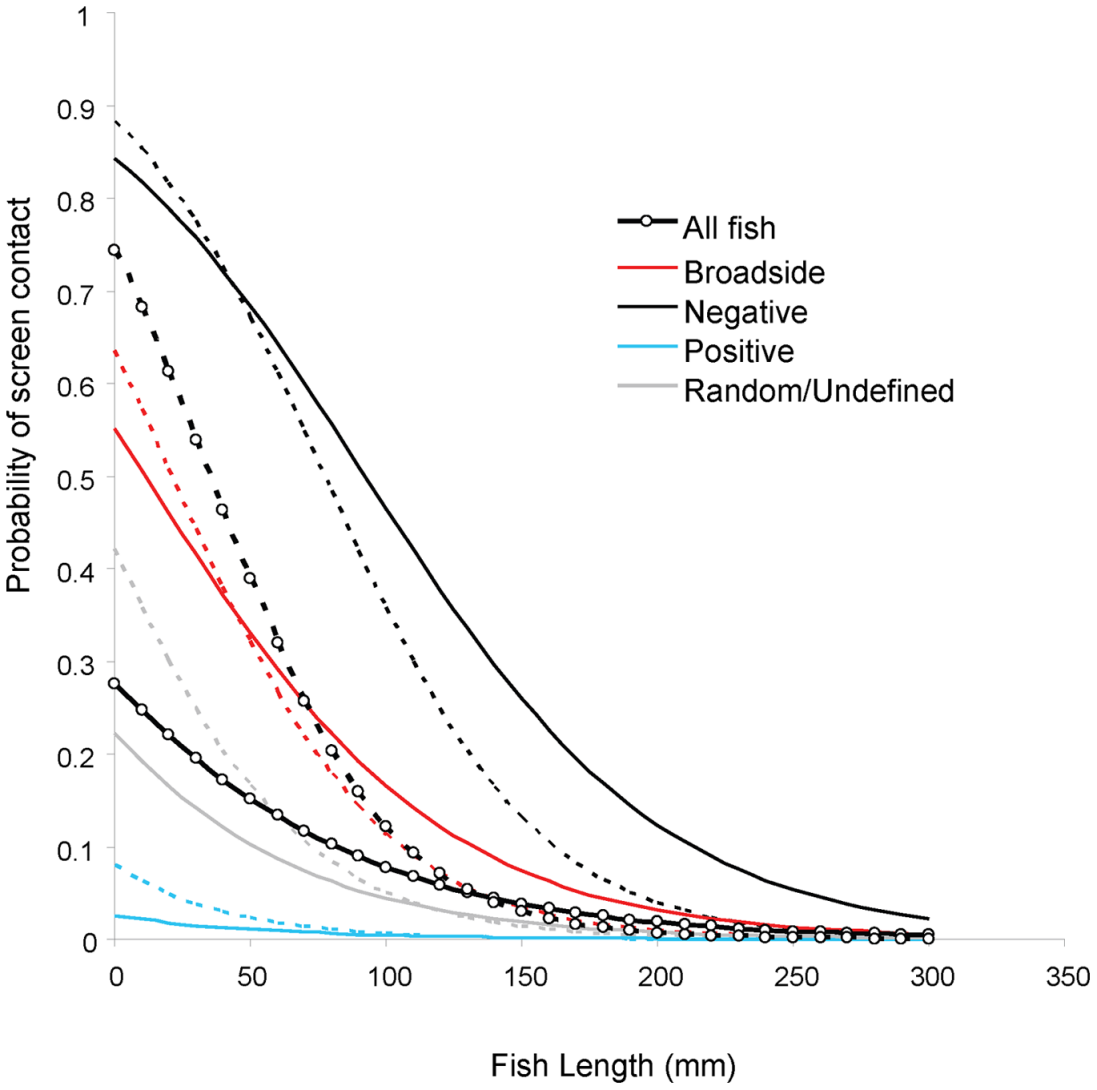
Predicted relationship of probability of screen contact with length and rheotaxis of fish (see methods for description of rheotactic behaviour). Solid lines are at 0.1 m.sec^−1^ and broken lines are at 0.5 m.sec^−1.^

The size range of fish contacting the screen was smaller than for those that avoided contact (Figure 3). Furthermore, the probability of screen contact increased with decreasing fish length (Figure 4). Fish below 150 mm were more likely to contact the screen as approach velocity increased from 0.1 to 0.5 m.sec^−1^. Fish smaller than 50 mm, had a 40 to 75% chance of contacting the screen when approach velocities were 0.5 m.sec^−1^, compared to 15 to 30% at 0.1 m.sec^−1^ (Figure 4). Rheotactic behaviour was significantly associated with the likelihood of screen contact at 0.1 m.sec^−1^ (χ^2^ = 226.7, df = 3, p<0.0001) and 0.5 m.sec^−1^ (χ^2^ = 48.1, df = 3, p<0.0001) (Figure 4). At both high and low approach velocities fish displaying negative rheotaxis were much more likely to contact the screen than those displaying positive rheotaxis (Figure 4 and Table 3). Negatively orientated fish were 19 times more likely to contact the screen at 0.1 m.sec^−1^ and eight times more likely at 0.5 m.sec^−1^ (Table 3). Positive aligned fish were significantly less likely to make contact (14 times less likely at 0.1 and 33 times less likely at 0.5 m.sec^−1^). Fish displaying broadside rheotaxis were four times more likely to make contact than those showing random orientation at 0.1 m.sec^−1^, but not different to random fish at 0.5 m.sec^−1^ (Table 3).

**Table 3:** Odds Ratios of rheotactic categories compared to random orientation.

	0.1 m.sec^−1^		0.5 m.sec^−1^	
Rheotaxis	Odds Ratio*	Comparison to random†	Odds Ratio*	Comparison to random†
Broadside	4.23	sig	1.22	ns
Negative	18.9	sig	7.81	sig
Positive	0.07	sig	0.03	sig

* The odds ratio is the increase in the probability of contact when compared to random orientation. For example, fish showing positive rheotaxis at the 0.1 m/s velocity are 14×less likely to make contact (1÷0.07 = 14).

† Significant (sig) or non-significant (ns) at the p = 0.05 level.

## Discussion

### Optimising Screen Design for the Murray-Darling Basin

Multiple species and size classes were susceptible to entrainment and screen contact at the approach velocities tested. Approach velocity should therefore be considered when developing generic criteria for screening programs in the Murray-Darling Basin. Higher velocities increased both the risk of contact and entrainment, and this was further influenced by fish size, with smaller fish substantially more susceptible. Although approach velocities specified for the protection of fish differ throughout the world, the ranges tested in our study matched those adopted in screening programs elsewhere. For example, approach velocities prescribed for the protection of juvenile anadromous salmonids in rivers range from 0.1 m.sec^−1^ (0.33 f.sec^−1^) for fry (<60 mm length) to 0.2 m.sec^−1^ (0.8 f.sec^−1^) for fingerlings (>60 mm) [13] Approach velocities of 0.15 m.sec^−1^ are seen as acceptable for juvenile northern pike (*Esox lucius*) [25] which are weaker swimmers than salmonids [37].

Susceptibility to contact or entrainment varied greatly throughout the fish assemblage. Smaller fish (<150 mm) were most vulnerable to screen contact and swimming behaviour appeared associated with likelihood of contact. The ability of a fish to avoid contact with a screen is therefore associated with its size and swimming ability, as well as behavioural response when exposed to an approach velocity. Laboratory studies of other species at simulated fish screens report similar findings, with there being a positive relationship between fish size and its ability to avoid contact or impingement (prolonged contact) [25] and a positive relationship between contact rate and approach velocity [31]. When encountering an approach velocity in front of a screen fish typically respond by swimming into the current (positive rheotaxis), with swimming speeds increasing with velocity [31], [35]. There are likely to be critical thresholds where the approach velocity will exceed the ability for a fish to effectively hold its position which is the point of contact for most species [35].

The maximum approach velocity tested here (0.5 m.sec^−1^) appeared sufficient to protect fish against entrainment. However, contact rates were significantly higher at 0.5 than at 0.1 for small fish (<150 mm). Screen contact or impingement may increase stress and injury to a fish [38], but others have shown that this may not be sufficient to impact on survival [25], [30], [31], [35]. Until the lethal and sub-lethal effects of screen contacts are better understood for Murray-Darling Basin species, it is advisable that approach velocities not exceed 0.1 m.sec^−1^, to ensure much lower contact rates for juveniles and fish smaller than 150 mm. As larval fish are also vulnerable to entrainment at diversions in large numbers [8], [9], [21], but were not studied here, further research into their survival at fish screens is also warranted.

Unscreened diversions in the Murray-Darling Basin have high fish entrainment rates [8]. Determining the optimal fish screen mesh size was assumed *a priori* to be important when mitigating potential impacts on fish. Mesh size, however, had little influence on entrainment rates when a woven mesh was used at both higher and lower approach velocities. The material from which a screen is constructed may therefore be less important than optimising approach velocity in mitigating entrainment. Comparisons of entrainment and survival rate of bull trout (*Salvelinus confluentus*) fry exposed to different screen materials support this assertion [27]. In that study, little difference in survival and entrainment rates were found between vertical profile bar, perforated plate, horizontal profile bar and woven-wire screen. It may be that most benefit can be gained from using a screen design which optimises approach velocity whilst minimising debris accumulation and flow restriction. Identifying a solution which satisfies all three of these criteria will ensure solutions are fish-friendly, require little maintenance and satisfy irrigation delivery needs [12].

### Effectiveness of a Dual Approach for Field Evaluations

The dual method of directly quantifying entrainment with nets and quantifying screen contact using Sonar had certain advantages. Sonar provided information on behaviour around screens which could not be gathered by netting alone. It is clear that the low entrainment rates determined by net catches were not due to the absence of fish around the experimental screen, because many fish were observed to approach the screen face. Although the majority of fish managed to avoided entrainment, many still made contact with the screen. As previously mentioned, it is still uncertain whether these contacts may need to be minimised to ensure the protection of smaller fish [38].

It was difficult to determine whether a fish swimming towards the water surface contacted the screen face, was impinged for a prolonged period, was entrained, or avoided contact all together by moving over the top of the screen. This is because images captured by the sonar are typically recorded in 2-dimensions, which can limit the ability to track target positions accurately. It is possible that this may have led to an over-estimation of contact rate and fish impact. However, experiments on golden perch exposed to equivalent approach velocities in a laboratory flume were found to produce slightly higher estimates of contact probability than determined in the current study using the sonar for an equivalent size range of fish (40–50 mm) [39]. This provides added confidence that, despite this potential limitation, field estimates using sonar have great potential for field verification of laboratory studies.

A common field-related constraint is the inability to control sample sizes of fish among treatments. The number of fish approaching the screen varied among sites and days (identified by the sonar, electrofishing and seine netting) and reduced the statistical power to detect differences. This limitation is commonly experienced in field-based studies and can be overcome to some degree by increasing sampling effort and the level of replication [40]. Finally, it was not possible to distinguish between species using the sonar method, making it difficult to ascertain whether certain species or life history stages were being preferentially impacted or protected. Species specific performance of screens may be best evaluated using lab-based methods as in [25], [35], [39].

The optimisation of fish screen criteria in the Murray-Darling Basin will need to be an ongoing activity, with criteria refined as more data become available. Based on the strengths and limitations of the field approach used here, it may be prudent not to use a field approach in isolation, but rather a combination of laboratory study with subsequent field validation. For field validation, we have demonstrated that a dual method using netting and sonar would be preferable to netting alone.

### Conclusion

No screening criteria exist for Australian freshwater fishes and this study has provided the first results from which to begin optimising screen design to mitigate the impact of irrigation diversions on fish populations in the Murray-Darling Basin. The results indicate that the design of fish screens in the Murray-Darling Basin should aim at minimising entrainment and screen contact by optimising approach velocities. Vertical panel screens generating velocities up to 0.5 m.sec^−1^ have great potential for reducing the entrainment of a wide range of species and size ranges of fish in the Murray-Darling Basin.

Small fish, however, did have a significantly higher probability of contacting the screen face at 0.5 m.sec^−1^ than 0.1 m.sec^−1^. Screens aimed at protecting a large proportion of the migratory assemblage need to be designed with the needs of the most susceptible species and size classes. The protection of a selection of size classes may not be adequate for the protection of populations. The extraction of even a small percentage of early life stage fish may represent a substantial loss of potential recruits from main river environments [18]. Failure to protect small juvenile fish could major implications for the sustainability of Murray-Darling fish populations, since it is felt that poor recruitment over several decades, rather than poor spawning, can be responsible for differences in fish faunas between rivers [3]. Until the severity and potential sub-lethal and lethal effects of contacts is better understood for Murray-Darling Basin fish species, it is recommended that a precautionary approach velocity of 0.1 m.sec^−1^ be applied where juvenile fish or those smaller than 150 mm require protection. Such an approach velocity is in line with the accepted standard for the protection of fish fry in other parts of the world (e.g. [13], [25]) and there are many screen designs already available which could meet these standards [12]. If approach velocity can be optimised, it would appear from this study that the aperture size of screen mesh may be a less important consideration for fish protection.

Although these findings are of direct importance to the sustainability of the irrigation sector, it is predicted that water abstraction not associated with cropping (domestic, industrial and livestock) will increase by about 50% by 2025 [41]. Flows are intrinsically linked with river health and it is crucial that river ecosystems are protected from current diversion practices and future expansion. Fish screens are an effective way of mitigating one impact of water diversion, but they need to be engineered in a way that affords the greatest protection for a greatest number of species. The approach outlined in this study appears to be a suitable way to refine screen design criteria in the wild, or field-validate laboratory results, thereby encompassing the entire assemblage of fish under actual riverine conditions.

## Materials and Methods

### Ethics Statement

All field studies outlined in this paper were authorised under a scientific research permit (permit No: P01/0059) issued by the NSW Department of Primary Industries under section 37 of the Fisheries Management Act 1994. This permit authorises the collection of fish in all waters of New South Wales. The river sites sampled were not privately owned or protected and no endangered or protected species were involved in this study. All fish collection was carried out in an ethical manner in accordance with Animal Research Authority 04/08 issued by NSW Primary Industries (Fisheries) Animal Care and Ethics Committee in compliance with the Animal Research Act 1985.

### Study Area

The study was undertaken in the Murray-Darling Basin, one of Australia’s largest river systems, occupying over 1 million km^2^ and contributing significantly to the country’s agricultural production. Rivers of the Murray-Darling Basin are among the most variable in flow in the world [42] and significant water resource development supports intensive dryland cropping. Collectively, several large-scale irrigation schemes and many smaller independent irrigators divert as much as 87% of flows from almost all of the Basin’s Rivers [43]. But abstraction at dams and weirs into canal systems can be substantially more at certain places and times, sometimes exceeding the volume of water released downstream [9], [44], [45].

There is mounting concern that fish losses at unscreened diversions in the Murray-Darling Basin is contributing to population declines [8], [9], [22]. Significant numbers of fish can be removed by pumps and regulators [8], [9]. Although the impact will undoubtedly differ between locations based upon local fish populations, time of year and size of diversion [9], [11], given the sheer volume of water that is diverted across the Murray-Darling Basin and the fact that at times the volumes diverted can significantly exceed that flowing downstream, it is likely that the numbers of fish removed are significant and will need to be reduced if fish population declines are to be addressed [22]. Currently programs to rehabilitate fish populations in the Murray-Darling Basin do not incorporate fish screening activities [46] and the development of fish screen criteria to support the initiation of screening programs is urgently required [12].

**Figure 5:**
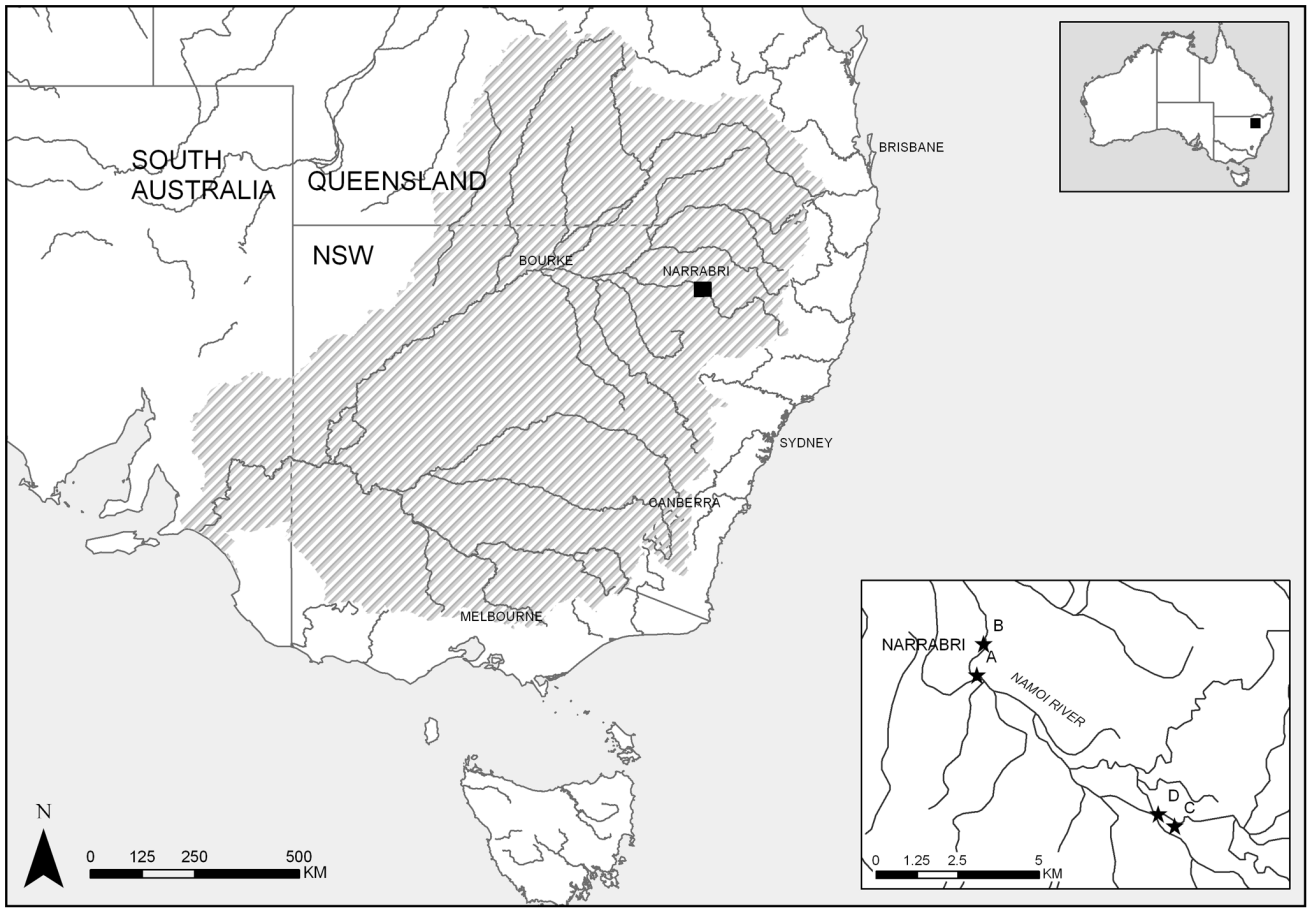
Location of the four study sites on the Namoi River showing the Murray-Darling Basin (grey thatched).

This study was undertaken between February and March 2011 at four sites, all within 12 km upstream of Narrabri (30.324963°S, 149.786742°E, 215 m elevation) on the Namoi River (New South Wales) (Figure 5). The Namoi River extends 845 km from the Great Dividing Range near Armidale to the Darling River and flow releases are heavily regulated during the irrigation season (between September and April) by two upland storages (Keepit and Split Rock Dams). Land use of the lower Namoi catchment consists predominately of irrigated wheat and cotton, as well as stock grazing. The system is characterised by low topography, deeply-incised channel banks and few instream regulating weirs. Water is typically pumped from the main channel into off-river storages, an approach common in most rivers in the northern Murray-Darling Basin [47]. Previous research has shown that significant numbers of fish can be entrained by irrigation pumps using this method of water extraction [8]. The fish assemblage of the lower Namoi River is of important conservation status, officially recognised as part of an endangered ecological community under the NSW Fisheries Management Act [48].

Field studies were undertaken at four sites, each with a gravel bar and heavy machinery access to place the pumping station. The river depth needed to be 2–3 m adjacent to the pump to ensure the screened intake was totally submerged and to permit screen mesh changes among treatments. River flow was relatively low at the time of study (0.03 m/sec ±0.01 S.D.), therefore the approach velocity created by the experimental screen was the dominant flow vector at the study sites.

### Experimental Pumping Station

**Figure 6:**
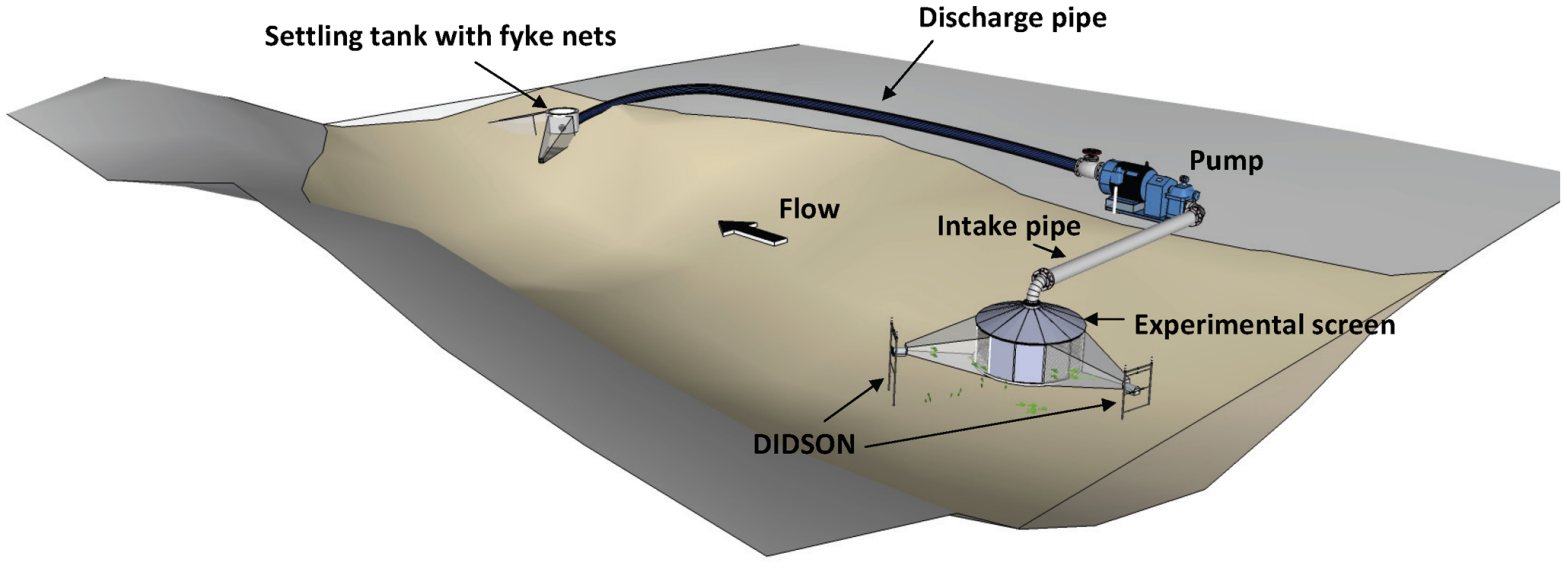
Schematic representation of the experimental pumping station showing major components. The experimental screen was cylindrical and comprised a series of removable panels to adjust approach velocity and mesh size. Fish that were entrained into the system travelled through the intake pipe, through the pump, along the lay-flat discharge pipe and were collected in fyke nets fitted to a settling tank. Fish behaviour in front of the screen face was quantified using DIDSON.

The experimental pumping station consisted of three main components: 1) a pump, 2) an intake pipe fitted with an experimental screen, and 3) a discharge outlet with fish collection nets (Figure 6).

A diesel-powered, mixed-flow (centrifugal) pump with a gear reduction marine drive was used to deliver water to the experimental facility. When operated at a very low head-differential between the intake and outlet (as in this study) the system was capable of up to 38 ML.day^−1^ which equates to a velocity of approximately 3 m.sec^−1^ through the 450 mm (18 inch) diameter intake pipe. A butterfly valve was used to control flow out of the discharge outlet. Flow rate, pipe velocity and total discharge obtained by the pumping station were measured during each trial with a Flo Pro (series 2) ultrasonic flow meter with a doppler sensor (Mace, Sydney Australia) installed in the intake pipe.

**Figure 7:**
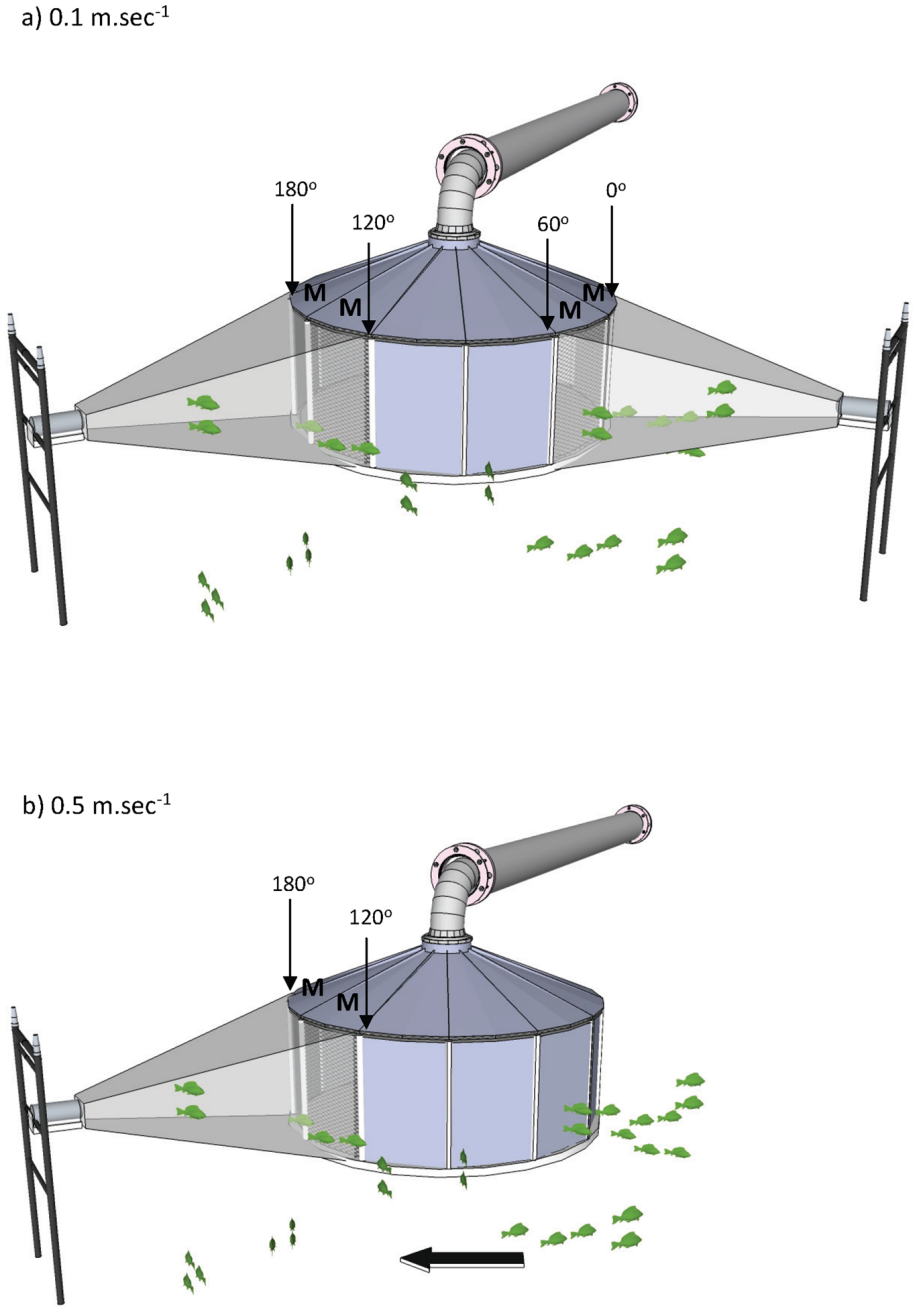
Diagram of the experimental fish screen showing position of mesh screen panels (M) and position of sonar for the a) 0.1 m.sec^−1^ approach velocity, b) 0.5 m.sec^−1^ approach velocity. Degrees are relative to 0° facing directly into downstream flow (indicated by the arrow). Mesh panels (M) of different aperture size could be interchanged or replaced with solid panels to manipulate approach velocity. To achieve the maximum velocity of 0.5 m.sec^−1^ all sides except two needed to be fitted with solid panels.

The 10 m long steel intake pipe was fitted with an experimental screen comprising of a solid flat bottom and a tapered top (2.4 m diameter and 1.5 m height: Figure 7). The screen outer surface incorporated 12 individual vertical panels (1 m high by 0.6 m wide), where screens of varying mesh size (5 mm, 10 mm and 20 mm woven galvanised wire) could be interchanged. Screen surface area was altered by replacing some mesh panels with solid aluminium blanks, thus closing off a proportion of the total diameter of the experimental screen to flow (Figure 7). Two approach velocities were tested in this study (0.13 m.sec^−1^±0.01 (SE) and 0.52 m.sec^−1^±0.02). These velocities were selected to correspond with screen design criteria applied in other parts of the world (e.g. [13]).

For the 0.5 m.sec^−1^ approach velocity, 10 solid panels were used, leaving two mesh panels as the open-screen face (0.36 m^2^ open-screen face, positioned 120–180° relative to the river flow: Figure 7). This position was on the open-water side (farthest from the bank) and pointing slightly downstream to maximise the possibility of encountering fish as they moved upstream. For the 0.1 m.sec^−1^ approach velocity, eight solid panels were used, leaving four panels as the open-screen face (0.72 m^2^ open-screen face, positioned 0–60° and 120–180° relative to the river flow: Figure 7) to create a larger surface area and hence a lower approach velocity. Approach velocity was measured at nine replicate points across (and 8 cm in front of) the screen face using a propeller-driven, digital flow meter (General Oceanics, Inc., Florida, U.S.A.), with pump discharge adjusted until the desired treatment approach velocities were obtained.

Diverted water was discharged back to the river downstream of the intake through a 40 m long, 480 mm (19 inch) heavy-duty vinyl ‘layflat’ irrigation hose (Figure 6). A settling tank was fitted to the end of the hose to reduce flow velocity and bed erosion, before the water was subsequently discharged into two fyke nets (6 mm stretch mesh, 10 m long, with two internal funnels). The nets were long enough to ensure that velocities had dissipated sufficiently to minimise injury to collected fish.

### Fish Assemblage Surveys

To establish what species the assemblage near the experimental screen were comprised of standardised electrofishing and seine netting surveys were conducted at the site after all pumping was concluded. Electrofishing was done with a five metre, twin-hulled aluminium boat mounted with a Smith-Root 7.5 GPP electrofishing unit using a pulsed (120 pulses per second) direct electrical current (DC). A total of 1080 electrofishing seconds (on time) was typically performed at each site across all available habitat in approximately a 200 m stretch of river. Electrofishing at each site was supplemented with three seine net samples (6 mm stretch mesh, 1 m drop, 10 m long). The net was deployed in a U-shape and pursed onto the shore. All fish collected in fyke nets following pump entrainment were measured to the nearest millimetre at the completion of the four hour experimental period. Measurements were taken from the tip of the snout to the tip of the tail in the mid-line (fork length for fork-tailed species and total length for rounded-tail species), which allowed for the comparison of length between net catches and those obtained from the sonar image. All fish were kept in an aerated live-well, then counted, measured and immediately released alive to ensure minimal distress.

### Statistical Analysis - Entrainment

The treatments were applied using a Latin Square experimental design [49] with separate squares for each approach velocity (Table 4). The effect of mesh size on catch per unit effort (CPUE: number of fish entrained per megalitre) was assessed using ANOVA after partitioning the effects of location and order of treatment. To compare overall differences in CPUE between velocities, a factorial ANOVA was fitted to the data pooled across (both) squares testing the factors velocity, mesh, order of treatment, location and the interaction between velocity and mesh.

**Table 4:** Tabular representation of the two Latin square experimental designs showing the order of allocation of mesh treatments within replicate runs for each of two approach velocities.

0.5 m.sec^−1^		Treatment mesh (mm)			
Order		1	2	3	4
Site	A	5	20	0	10
	B	20	10	5	0
	C	10	0	20	5
	D	0	5	10	20
**0.1 m.sec^−1^**		**Treatment mesh (mm)**			
Site	A	20	10	5	0
	B	10	0	20	5
	C	0	5	10	20
	D	5	20	0	10

### Quantifying Screen Contact

#### Acoustic image acquisition

Dual-frequency identification sonar (DIDSON; Sound Metrics Corp.) was used to quantify the number and nature of fish interactions with the experimental screen. The DIDSON was operated in high frequency mode (1.8 MHz), which generates near-video quality images over small distances (<12 m) [32]. DIDSON uses 96 beams to generate a total field of view of 29° horizontal by 14° vertical under high frequencies [50]. The sonar was horizontally mounted approximately 4 m from the screen face and 1 m below the water surface. The field of view allowed for a maximum of two screen panels to be observed by a single unit. Therefore one sonar was required for the higher velocity treatment and two sonar were simultaneously used for the lower velocity treatment when four panels of screen were required (Figure 7). Because the sonar units were not synchronised [51], there was some acoustic feedback and loss of picture quality but not of sufficient amount to hinder treatment comparison. The configuration however, did give acoustic images of adequate resolution to allow an observer to classify data on the basis of fish size and behaviour, but not species.

#### Post-processing and data analysis

Echograms from each sonar sample were captured using Sound Metrics topside software version 5.25 [52]. To expedite analysis, sub-sampling of each echogram was performed where the first 20 minutes of footage was discarded to account for any fish behavioural responses to pump start-up. A random start point was then selected in the next 10 minute interval and one minute of footage (600 frames) was extracted at each subsequent 10 minute interval (resulting in 22 independent one minute sub-samples for viewing).

**Figure 8:**
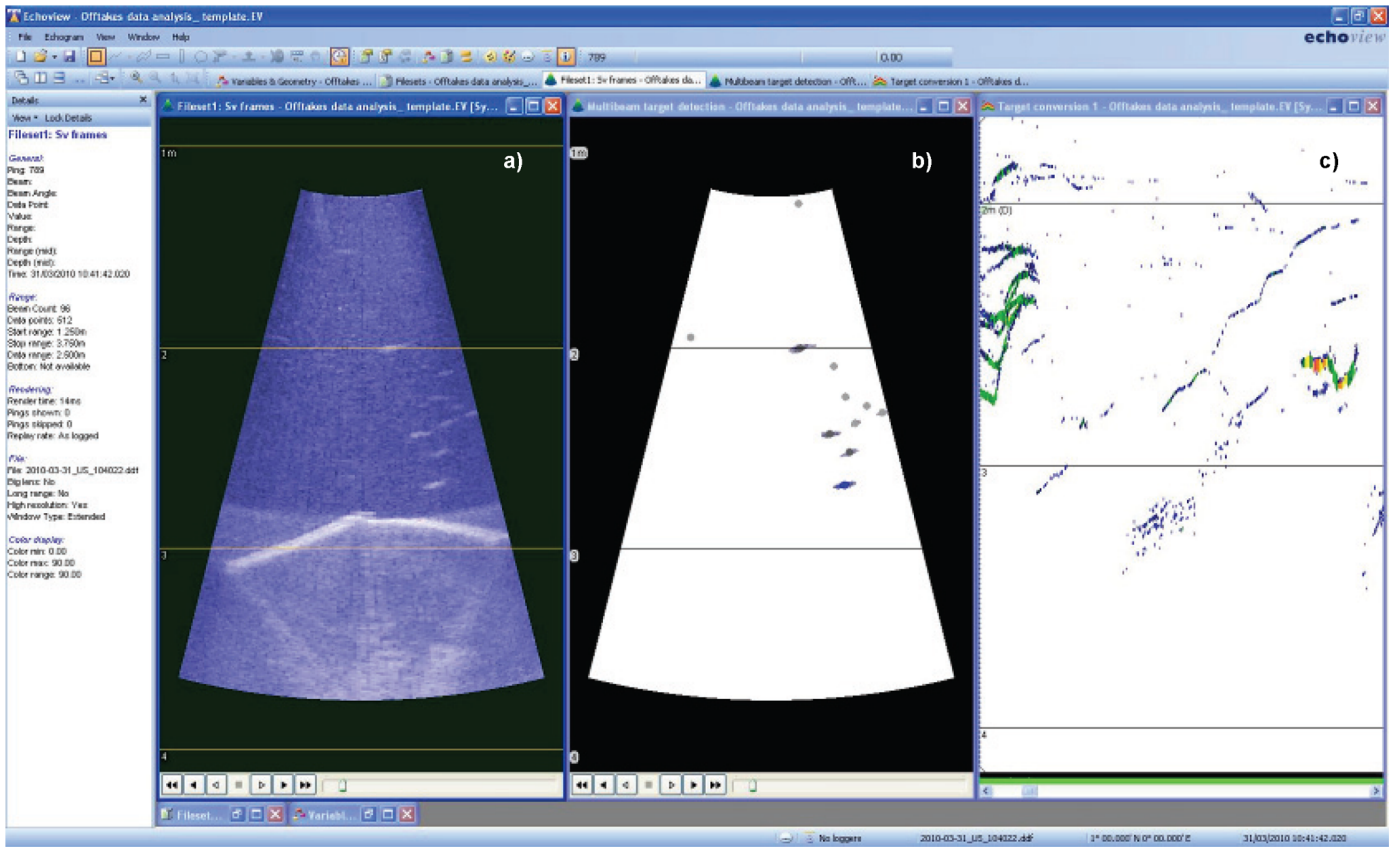
Screenshot showing acoustic echogram obtained from sonar (a) alongside post-processing modules created in Echoview, including a background reduction and target identification module (b) and fish tracking module (c). Screen panels can be seen as light coloured bands at the image bottom.

Post-processing modules were developed in Echoview (Myriax Software: [53]) and run simultaneously with the original echogram to assist in target identification, size measurements, trajectory and to classify movements in relation to the approach velocity (Figure 8). Firstly a background subtraction and target identification module was developed. To assist in recognising fish, debris and static objects (non-moving objects such as substrate and screen) were filtered from the echogram and only moving targets greater than 3 mm long were identified. Three millimetres was used as a maximum for exclusion to minimise the risk of filtering actual fish. The second module developed was for fish tracking to log positional data within the echogram and assist with target recognition and directionality. Analysing data in this way allowed determination of whether fish were approaching the screen or moving away from it.

A target area 0.5 m radius from the centre of each of the 2 panels was drawn on the echogram viewing window and only fish that entered this target area were deemed to have approached the screen. It is important to note that we refrain from using the term ‘impingement’ in this study and instead refer to screen contact. Impingement is typically used to refer to a prolonged (e.g. >3 second [25]) screen contact. The sonar was limited in its ability to discriminate a prolonged impingement from a contact due to acoustic feedback from the metal screen and a lack of resolution. Targets therefore became virtually invisible once touching the screen so the actual duration of contact could not be accurately determined. For the purposes of this study we subsequently refer to any screen interactions as contact, rather than impingement.

To assess contact probability, fish entering the field of view were measured (using an Echoview module) and then assigned to one of three behavioural categories: 1) contact – the fish entered the target area, took a path towards the screen and disappeared upon reaching it; 2) non-contact – the fish entered and then left the target area without touching the screen; or 3) not defined – the fish entered the target area although disappeared before either touching the screen or leaving the target area again.

It was deemed important to determine the swimming behaviour of individual fish in front of the screen as it provided some insight as to whether a certain size class of fish was attempting (but unable) to avoid contact due to excessive velocity. Rheotactic alignment in relation to the approach velocity vector was subsequently quantified for all fish in the field of view. Individual fish were assigned to one of the following five categories: 1) positive rheotaxis – the fish turned to face away from the screen swimming into the oncoming current; 2) negative rheotaxis – the fish moved head first towards the screen, or in the direction of the current; 3) broadside rheotaxis – the fish moved laterally (across) the current; 4) random – a combination of one or more rheotactic behaviours; or 5) not defined – no rheotactic alignment could be confidently defined.

### Statistical Analysis - Contact

The probability of contact (proportion of observed fish that contacted the screen) between the 0.1 and 0.5 m.sec^−1^ velocities was compared across all fish in each Latin square design using the log-likelihood ratio test [54]. Further, within each velocity treatment, a multiple logistic regression model [54] was fitted using the Latin square design to compare the contact probability between mesh types after partitioning the differences between sites and treatment order. Follow-up analysis compared the parameter estimates and odds ratios for each mesh size with those of the ‘no mesh’ treatment (Table 2). The odds ratio can be described as “the probability of an event occurring expressed relative to the probability of an event not occurring” [54]. In our models we define an event as screen contact.

To investigate whether the probability of contact was associated with fish length or rheotactic behaviour, a logistic model was used that added length of fish (mm), rheotactic alignment and potential interactions as covariates to the Latin square factors mesh size, location and order of treatment. Differences in the probability of contact between non-random and random rheotaxis was compared using the profile likelihood confidence intervals of the odds ratios [55]. The predicted probability of contact was plotted for fish of 0–300 mm length in each rheotactic category at each velocity.
